# Two-year longitudinal associations between nutritional status and frailty in community-dwelling older adults: Korean Frailty and Aging Cohort Study

**DOI:** 10.1186/s12877-023-03903-4

**Published:** 2023-04-05

**Authors:** Namhee Kim, Gwang Suk Kim, Chang Won Won, Jae Jun Lee, Min Kyung Park, Jinhee Shin, Miji Kim

**Affiliations:** 1 Wonju College of Nursing, Yonsei University, Wonju, Republic of Korea; 2 Mo-Im Kim Nursing Research Institute, College of Nursing, Yonsei University, 50-1, Yonsei-Ro, Seodaemun-Gu, Seoul, 03722 Republic of Korea; 3 Elderly Frailty Research Center, Department of Family Medicine, College of Medicine, Kyung Hee University, 26, Kyungheedae-Ro, Dongdaemun-Gu, Seoul, 02447 Republic of Korea; 4 Department of Nursing, Graduate School of Yonsei University, Seoul, Republic of Korea; 5 College of Nursing, Woosuk University, Jeollabuk-Do, Republic of Korea; 6 Department of Biomedical Science and Technology, East-West Medical Research Institute, College of Medicine, Kyung Hee University, Seoul, Republic of Korea

**Keywords:** Aged, Anorexia, Cohort studies, Frailty, Malnutrition, Nutrition assessment

## Abstract

**Background:**

Korea is expected to become a super-aged society in 2026, and improving nutritional status, which is directly related to health problems, is therefore important for increasing healthy life expectancy. Frailty is the most complex phenotype of aging, and leads to adverse health outcomes, disability, poor quality of life, hospitalization, and mortality. Malnutrition is a major risk factor for frailty syndrome. This study aimed to investigate the incidence of pre-frailty or frailty in the second wave (T2, 2018–2019) according to general characteristics and nutritional status in the first wave (T1, 2016–2017); and examine the longitudinal association of nutritional status in T1 and the incidence of pre-frailty or frailty in T2 among older adults living in a community.

**Methods:**

A secondary data analysis was performed using the Korean Frailty and Aging Cohort Study (KFACS). Participants comprised 1125 community-dwelling older Korean adults aged 70–84 years (mean age: 75.03 ± 3.56 years; 53.8% males). Frailty was assessed using the Fried frailty index, and nutritional status was assessed using the Korean version of the Mini Nutritional Assessment Short-Form and blood nutritional biomarkers. Binary logistic regression was used to identify longitudinal associations between the nutritional status at T1 and pre-frailty or frailty at T2.

**Results:**

Over the two-year follow-up period, 32.9% and 1.7% of the participants became pre-frail and frail, respectively. After the potential confounders were adjusted (sociodemographic, health behaviors, and health status characteristics), pre-frailty or frailty had a significant longitudinal association with severe anorexia (adjusted odds ratio [AOR], 4.17; 95% confidence interval [CI], 1.05–16.54), moderate anorexia (AOR, 2.31; 95% CI, 1.46–3.64), psychological stress or acute disease (AOR, 2.61; 95% CI, 1.26–5.39), and body mass index (BMI) less than 19 (AOR, 4.11; 95% CI, 1.20–14.04).

**Conclusions:**

Anorexia, psychological stress, acute disease, and low BMI are the most significant longitudinal risk factors for pre-frailty or frailty in older adults. As nutritional risk factors may be preventable or modifiable, it is important to develop interventions targeting the same. Community-based health professionals in health-related fields should recognize and manage these indicators appropriately to prevent frailty among older adults living in the community.

**Supplementary Information:**

The online version contains supplementary material available at 10.1186/s12877-023-03903-4.

## Background

According to the World Health Organization, maintaining proper nutritional status is one of the prerequisites for the well-being and “healthy aging” of older adults [1, 2]. Malnutrition, defined as undernourishment and deficient energy, frequently affects older adults [3, 4]. It is characterized by loss of muscle mass and weight [3, 4] and carries severe negative health outcomes [1]. Malnutrition accelerates age-associated changes, leading to loss of strength and muscle and increased sarcopenia [1, 3]. Malnutrition is a major risk factor associated with frailty syndrome [3] and is central to the phenotypic criteria for frailty [5].

Frailty is a multidimensional geriatric syndrome [5] that is related to adverse health outcomes, disability, poor quality of life, hospitalization, and mortality over time [6, 7]. It is recognized to be a reversible condition; researchers have proposed that early detection of frailty-related risk factors reduces its incidence [8]. Therefore, it is importance to assess nutrition and frailty among older adults, and develop consequent interventions [8, 9]. Exploratory research is needed to better understand the association between nutritional status and frailty, and to contribute to the successful management of these two geriatric characteristics.

Globally, malnutrition and frailty among community-dwelling older adults are becoming increasingly prevalent [9–12]. A systematic literature review exploring malnutrition and frailty among older adults living in the community showed that two out of three malnourished older adults were physically frail [12]. However, previous studies confirming the association between nutrition and frailty are controversial and have limitations in explaining causal relationships because of their cross-sectional design [13, 14]. Very few longitudinal studies have confirmed the causal association between nutritional status and frailty [15, 16]. Specific longitudinal risk factors for nutritional status should be identified in order to develop frailty preventative measures including nutritional assessments and interventions. Additionally, most studies have focused on older adults with disabilities in institutions and hospitals rather than those living in communities with larger populations and where early prevention should be given greater attention [14, 17]. Therefore, this study investigated whether specific nutritional status factors were longitudinally associated with frailty in community-dwelling older adults.

The aims of this longitudinal study were 1) to investigate the incidence of pre-frailty or frailty at follow-up according to general characteristics and nutritional status at the baseline and 2) to identify specific longitudinal factors associated with nutritional status at the baseline and the incidence of pre-frailty or frailty at follow-up in community-dwelling older adults enrolled in the nationwide Korean Frailty and Aging Cohort Study (KFACS).

## Methods

### Design and study population

**Fig. 1:**
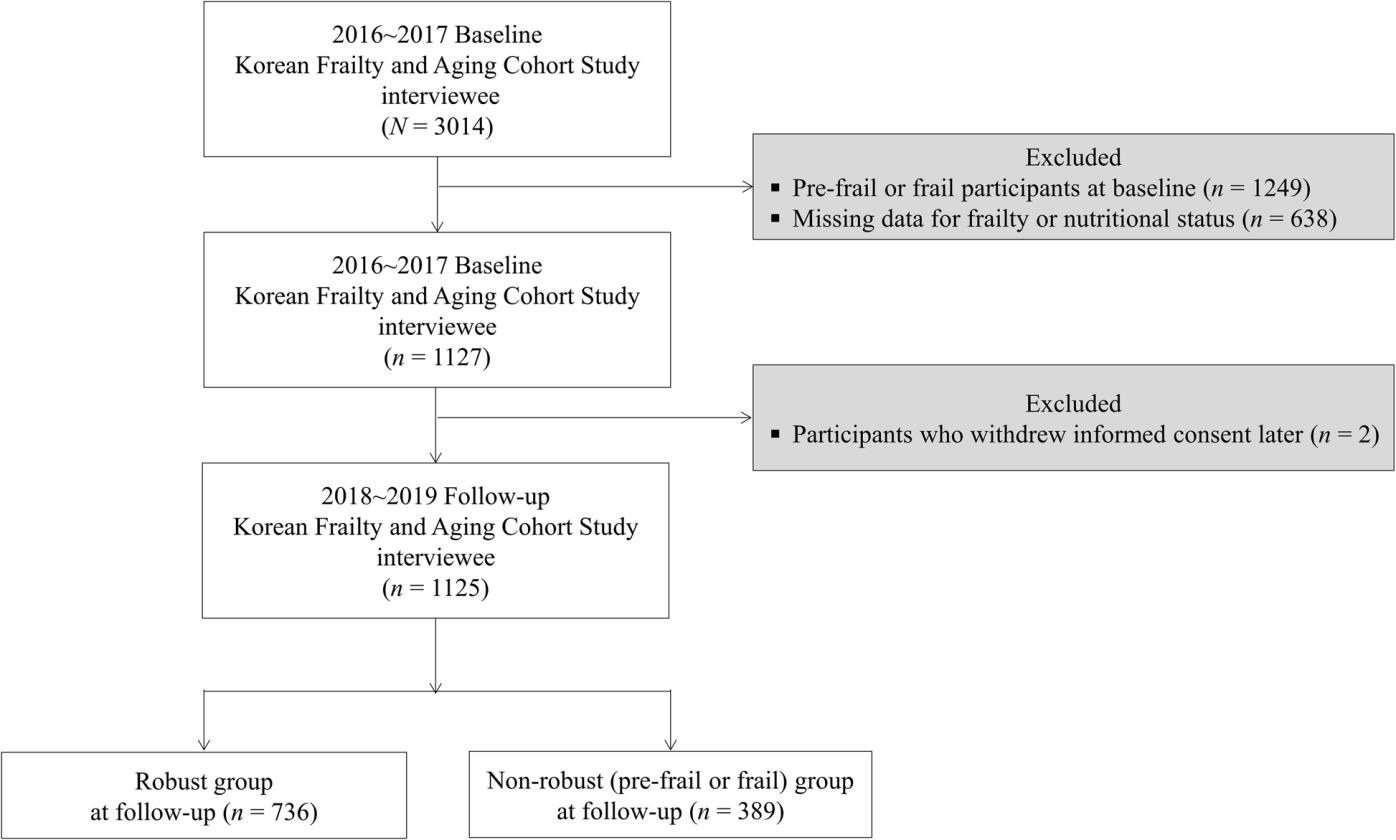
Flow chart of the participants

This study was based on data from the first (T1, baseline period in 2016–2017) and second (T2, two-year follow-up period in 2018–2019) waves of the KFACS, a multicenter longitudinal study covering 10 centers across rural, suburban, and urban regions in Korea [18, 19]. This cohort study has been followed every two years [18, 19]. Recently, the third wave (T3, 2020–2021) was conducted. However, the data has not been released. KFACS participants were recruited by each center and included older adults aged 70–84 years, stratified according to age (70–74, 75–79, and 80–84 years, 6:5:4 ratio) and sex [18, 19]. The age range reflects the frailty consensus’s proposal that all older adults aged ≥ 70 years be screened for frailty [7, 19]. T1 of the KFACS included 3014 older adults [18, 19]. To identify new cases of pre-frail or frail at T2 among robust older adults at T1, this study included only robust older adults at T1. Exclusion criteria were older adults who were pre-frail or frail at T1 (*n* = 1249), those with missing data related to frailty (*n* = 106) or nutritional status (*n* = 532), and those who withdrew informed consent later (*n* = 2). The final sample size selected for this study was 1125 older adults who were robust at T1 (Fig. 1).

### Measurements

#### Frailty

Frailty was assessed at T2 using the Fried Frailty Phenotype with a modified cutoff point [5, 19, 20]. The index consists of five domains [19]: 1) Unintentional weight loss was defined as a loss of 4.5 kg, or more than 5% of the total body weight in the last year. 2) Weakness was measured by the maximal grip strength in kg after measuring twice for each hand using a hand grip dynamometer (T.K.K.5401, Takei Scientific Inc. Co., Ltd., Japan). Weakness was defined as the 20^th^ percentile of grip strength stratified by gender and body mass index quartile. 3) Exhaustion was measured using two self-reported questions from the Center for Epidemiological Studies-Depression scale: “I felt that everything I did was an effort” and “I could not get going.” If the answer was yes to either of these questions for three or more days in a week, it was categorized as exhaustion. 4) Walking speed was measured by a 4-m gait speed test using an automatic timer (Gaitspeedometer, Dyphi, Daejeon, Korea) with an acceleration and deceleration phase of 1.5 m. The mean values were selected after measuring the variables twice and the lowest 20% of gait speed stratified by gender and height was suggested as a cut-off. 5) Low physical activity was measured using the International Physical Activity Questionnaire and was calculated using a metabolic equivalent task. Low physical activity was defined as a person with less than 20% of total energy consumed for a week based on the 2008 Korea Elderly Survey [19–22]. Each domain was scored by 0 or 1, and the summed scores range from 0 to 5. A score of 0 was considered robust, 1–2 indicated pre-frailty, and 3–5 indicated frailty [5, 19]. Based on the scores, we designated two groups, robust and non-robust (i.e., pre-frail or frail), for this study.

#### Nutritional status

Nutritional status was assessed at T1 using blood nutritional biomarkers and the Korean version of the Mini Nutritional Assessment Short-Form (MNA-SF) [23], a widely used, validated, and established nutrition screening scale. The MNA-SF consists of six items [23]: 1) Anorexia was assessed using the self-reported question about decreased food intake due to loss of appetite, digestive problems, and chewing or swallowing difficulties in the past three months. It was categorized into three groups: no decrease, moderate, and severe. 2) Weight loss in the past three months was categorized into four groups: no change, 1‒3 kg loss, does not know, and > 3 kg loss. 3) Mobility was measured using a question about activity and was categorized into three groups: goes out, able to get out of bed/chair but does not go out, and bed or chair bound. 4) Experience of psychological stress or acute disease in the past three months was categorized into two groups: yes and no. 5) Presence of neuropsychological problems was categorized into three groups: no problems, mild dementia, and severe dementia or depression. 6) BMI was categorized into four groups: BMI ≥ 23, 21 ≤ BMI < 23, 19 ≤ BMI < 21, and BMI < 19. Each item was weighted according to the response. The summed weighted scores range from 0 to 14. A score of 12‒14 indicates normal nutritional status, 8‒11 indicates a risk of malnutrition, and 0‒7 indicates malnutrition. Blood nutritional biomarkers included hemoglobin, albumin, total protein, and total cholesterol levels.

#### Confounders

Based on a literature review, this study included three confounding factors (sociodemographic, health behaviors, and health status characteristics) previously self-reported as general characteristics from T1 [19, 22, 24]. Sociodemographic characteristics included age, gender, educational level, marital status, living area, and living arrangement. Health behavioral characteristics included smoking, drinking, and sleep. Health status characteristics included activities of daily living (ADL) dependency, instrumental activities of daily living (IADL) dependency, cognitive function, oral health, and comorbidity. ADL/IADL dependency was assessed using the Korean ADL/IADL scale [24]. Cognitive function was assessed using the Korean version of the Mini-Mental State Examination, and was defined as either low (≤ 23) or normal (> 24) [19]. Oral health was categorized into three groups—poor, fair, and good—based on a question regarding chewing difficulties [19, 22]: “Are you currently experiencing discomfort when chewing food due to problems in your mouth, such as teeth, dentures, or gums?” The number of comorbidities was estimated from the total number of self-reports and the current medical history diagnosed by a physician [19, 22].

### Statistical analysis

Descriptive statistics were presented for all variables, and the results were presented as numbers (%) for categorical variables. Chi-squared tests or Fisher’s exact tests were performed to examine differences in the general characteristics of participants, their nutritional status, and frailty. The cumulative incidence of pre-frailty or frailty was determined from follow-up visits in the longitudinal analysis. A binary logistic regression was performed to calculate the odds ratios (ORs) with 95% confidence intervals (CI) of longitudinal associations between nutritional status at T1 and frailty at T2, adjusting for potential confounders. All analyses were performed using SPSS 28.0 software (IBM, Inc., Chicago, IL), and *p*-values < 0.05 were considered significant.

## Results

### Baseline participants’ general characteristics and nutritional status

Tables 1 and 2 summarize the participants’ general characteristics and nutritional status at T1, grouped by their frailty status at T2. At T1, the participants’ average age was 75.03 ± 3.56 years, and 53.8% were male. At T1, based on participants’ nutritional status, 90.4% had normal nutrition, 9.4% were at risk of malnutrition, and 0.2% were malnourished.

**Table 1:** Baseline participants’ general characteristics by follow-up frailty phenotype (*N* = 1125)

Variables	Total	Robust	Non-robust (pre-frail or frail)	*x*^*2*^	*p*
**Total *N* (%)**	**1125 (100.0)**	**736 (65.4)**	**389 (34.6)**		
**Sociodemographic Characteristics**					
Age					
70–74	540 (48.0)	376 (69.6)	164 (30.4)	8.126	.005
75–84	585 (52.0)	360 (61.5)	225 (38.5)		
Gender					
Male	605 (53.8)	423 (69.9)	182 (30.1)	11.691	.001
Female	520 (46.2)	313 (60.2)	207 (39.8)		
Education level					
Uneducated, village school	119 (10.6)	70 (58.8)	49 (41.2)	9.971	.007
Elementary, middle, high school	738 (65.7)	471 (63.8)	267 (36.2)		
College	267 (23.7)	195 (73.0)	72 (27.0)		
Marital status					
Bereaved, divorced, separated, not married	300 (26.7)	174 (58.0)	126 (42.0)	10.131	.002
Married	824 (73.3)	562 (68.2)	262 (31.8)		
Living area					
Rural	253 (22.6)	136 (53.8)	117 (46.2)	19.218	< .001
Suburban, urban	865 (77.4)	594 (68.7)	271 (31.3)		
Living arrangement					
Living alone	206 (18.3)	125 (60.7)	81 (39.3)	2.507	.124
Living with others	919 (81.7)	611 (66.5)	308 (33.5)		
**Health Behaviors Characteristics**					
Smoking					
Never/Past smoker	1061 (94.6)	700 (66.0)	361 (34.0)	2.177	.163
Current smoker	60 (5.4)	34 (56.7)	26 (43.3)		
Drinking					
Never/Past drinker	509 (45.4)	309 (60.7)	200 (39.3)	9.384	.002
Current drinker	612 (54.6)	425 (69.4)	187 (30.6)		
Sleep					
< 6 h	363 (32.3)	233 (64.2)	130 (35.8)	.522	.774
6-8 h	741 (65.9)	490 (66.1)	251 (33.9)		
≥ 9 h	21 (1.8)	13 (61.9)	8 (38.1)		
**Health Status Characteristics**					
ADL dependency					
No	1044 (92.8)	688 (65.9)	356 (34.1)	1.466	.275
Yes	81 (7.2)	48 (59.3)	33 (40.7)		
IADL dependency					
No	728 (64.8)	479 (65.8)	249 (34.2)	.110	.743
Yes	395 (35.2)	256 (64.8)	139 (35.2)		
Cognitive function					
Low cognition	120 (10.7)	70 (58.3)	50 (41.7)	2.984	.085
Normal cognition	1005 (89.3)	666 (66.3)	339 (33.7)		
Oral health					
Poor	339 (30.1)	199 (58.7)	140 (41.3)	14.087	.001
Fair	100 (8.9)	59 (59.0)	41 (41.0)		
Good	686 (61.0)	478 (69.7)	208 (30.3)		
Comorbidity					
0	176 (16.1)	126 (71.6)	50 (28.4)	10.877	.004
1	283 (25.8)	199 (70.3)	84 (29.7)		
≥ 2	637 (58.1)	390 (61.2)	247 (38.8)		

Non-responses were excluded from the analysis. Values are presented as number (%)

*ADL* Activities of daily living, *IADL* Instrumental activities of daily living

**Table 2:** Baseline participants’ nutritional status by follow-up frailty phenotype (*N* = 1125)

Variables	Total	Robust	Non-robust (pre-frail or frail)	*x*^*2*^	*p*
**Total *N* (%)**	**1125 (100.0)**	**736 (65.4)**	**389 (34.6)**		
**MNA Screening**					
Malnutrition	2 (0.2)	1 (50.0)	1 (50.0)	2.435^a^	.294
At risk of malnutrition	106 (9.4)	63 (59.4)	43 (40.6)		
Normal nutrition	1017 (90.4)	672 (66.1)	345 (33.9)		
**MNA Screening Items**					
Anorexia					
Severe	12 (1.1)	5 (41.7)	7 (58.3)	18.453^a^	< .001
Moderate	125 (11.1)	62 (49.6)	63 (50.4)		
No	988 (87.8)	669 (67.7)	319 (32.3)		
Weight loss					
> 3 kg weight loss	6 (0.5)	4 (66.7)	2 (33.3)	.572^a^	.932
Unknown	10 (0.9)	7 (70.0)	3 (30.0)		
1-3 kg weight loss	124 (11.0)	78 (62.9)	46 (37.1)		
No	985 (87.6)	647 (65.7)	338 (34.3)		
Mobility					
Bed or chair bound	0 (0.0)	0 (0.0)	0 (0.0)	-^b^	-^b^
Able to get out of bed/chair but does not go out	0 (0.0)	0 (0.0)	0 (0.0)		
Goes out	1125 (100.0)	736 (65.4)	389 (34.6)		
Psychological stress or acute disease					
Yes	63 (5.6)	33 (52.4)	30 (47.6)	5.017	.029
No	1062 (94.4)	703 (66.2)	359 (33.8)		
Neuropsychological problems					
Severe dementia or depression	8 (0.7)	4 (50.0)	4 (50.0)	2.746^a^	.246
Mild dementia	1 (0.1)	0 (0.0)	1 (100.0)		
No	1116 (99.2)	732 (65.6)	384 (34.4)		
BMI (kg/m^2^)					
BMI < 19	21 (1.9)	11 (52.4)	10 (47.6)	2.414	.494
19 ≤ BMI < 21	89 (7.9)	61 (68.5)	28 (31.5)		
21 ≤ BMI < 23	241 (21.4)	162 (67.2)	79 (32.8)		
BMI ≥ 23	774 (68.8)	502 (64.9)	272 (35.1)		
**Blood Nutritional Biomarkers**					
Hemoglobin					
Abnormal	151 (13.4)	89 (58.9)	62 (41.1)	3.239	.081
Normal	974 (86.6)	647 (66.4)	327 (33.6)		
Albumin					
Abnormal	2 (0.2)	1 (50.0)	1 (50.0)	.211^a^	1.000
Normal	1123 (99.8)	735 (65.4)	388 (34.6)		
Total protein					
Abnormal	102 (9.1)	60 (58.8)	42 (41.2)	2.159	.156
Normal	1023 (90.9)	676 (66.1)	347 (33.9)		
Total cholesterol					
Abnormal	276 (24.5)	185 (67.0)	91 (33.3)	.417	.560
Normal	849 (75.5)	551 (64.9)	298 (35.1)		

Non-responses were excluded from the analysis. Values are presented as number (%). Normal range for hemoglobin 13.0–17.5 g/dL (male), 12.0–16.0 g/dL (female); albumin 3.5–5.2 g/dL; total protein 6.6–8.7 g/dL; total cholesterol < 200 mg/dL

*BMI* Body mass index, *MNA* Mini Nutritional Assessment

^a^Fisher exact test

^b^Analysis is not possible because there are no participant

### Incidence of pre-frailty or frailty at follow-up according to general characteristics and nutritional status at baseline

Over the two-year follow-up period, 32.9% and 1.7% of participants became pre-frail and frail, respectively. The incidence of pre-frailty or frailty was higher in older females, those with lower education levels, single marital status (bereaved, divorced, separated, and not married), living in rural living areas, never or past drinkers, having poor or fair oral health, and two or more comorbidities (all *p* < 0.01, Table 1). Malnutrition or risk of malnutrition as per the MNA was not associated with incidence of pre-frailty or frailty two years later. However, the incidence of pre-frailty or frailty was higher in older adults with severe or moderate anorexia, psychological stress, or acute disease (all *p* < 0.05, Table 2). Supplementary Material Figure S1 shows the incidence of pre-frailty or frailty at T2 according to the nutritional status screening items at T1. Over the two-year follow-up period, the incidence of pre-frailty or frailty was 50.4% and 58.3% among older adults at T1 with moderate and severe anorexia, respectively. In addition, the incidence of pre-frailty or frailty was 33.8% and 47.6% in older adults without and with psychological stress or acute disease at T1, respectively.

### Longitudinal association between nutritional status and frailty

Table 3 shows the results of binary logistic regression between nutritional status at T1 and frailty at T2, with the robust group as the reference (Cox and Snell R^2^, 0.10; Nagelkerke R^2^, 0.14). After adjusting for potential confounding factors of general characteristics, severe (adjusted odds ratio [AOR], 4.17; 95% confidence interval [CI], 1.05–16.54) and moderate (AOR, 2.31; 95% CI, 1.46–3.64) anorexia were found to be significant predictors of pre-frailty or frailty, as were psychological stress or acute disease (AOR, 2.61; 95% CI, 1.26–5.39), and a BMI less than 19 (AOR, 4.11; 95% CI, 1.20–14.04).

**Table 3:** Longitudinal association between nutritional status at baseline and frailty at follow-up (*N* = 1125)^a^

Variables	Non-robust (pre-frail or frail) (ref. Robust)	
	**Adjusted OR**	**95% CI**
**MNA Screening** (ref. Normal nutrition)		
Malnurition	0.05	0.00‒1.58
At risk of malnutrition	0.46	0.21‒1.02
**MNA Screening Items**		
Anorexia (ref. No)		
Severe	4.17^*^	1.05‒16.54
Moderate	2.31^***^	1.46‒3.64
Weight loss (ref. No)		
> 3 kg weight loss	2.23	0.25‒19.83
Unknown	0.83	0.19‒3.64
1-3 kg weight loss	0.93	0.58‒1.49
Mobility (ref. Goes out)		
Bed or chair bound	-^c^	-^c^
Able to get out of bed/chair but does not go out	-^c^	-^c^
Psychological stress or acute disease (ref. No)^b^		
Yes	2.61^*^	1.26‒5.39
Neuropsychological problems (ref. No)^b^		
Mild dementia/Severe dementia or depression	2.75	0.67‒11.30
BMI (kg/m^2^) (ref. BMI ≥ 23)		
BMI < 19	4.11^*^	1.20‒14.04
19 ≤ BMI < 21	0.99	0.56‒1.74
21 ≤ BMI < 23	0.95	0.67‒1.33
**Blood Nutritional Biomarkers** (ref. Normal)^b^		
Abnormal hemoglobin	1.40	0.95‒2.06
Abnormal albumin	0.80	0.04‒14.76
Abnormal total protein	1.33	0.85‒2.08
Abnormal total cholesterol	1.16	0.84‒1.62
**Cox** **and Snell** **R**^**2**^	0.10	
**Nagelkerke** **R**^**2**^	0.14	

Non-responses were excluded from the analysis. Values are adjusted odds ratio (95% confidence interval)

*BMI* Body mass index, *CI* Confidence interval, *MNA* Mini Nutritional Assessment, *OR* Odds ratio

^a^Binary logistic regression test. Sociodemographic, health behaviors, and health status variables were all adjusted. The references for the dependent variable was robust

^b^Dichotomized variables

^c^Analysis is not possible because there are no participant

^*^p < .05, ^**^p < .01, ^***^p < .001

## Discussion

This study examined the incidence of pre-frailty or frailty over a two-year follow-up period and confirmed the longitudinal association between specific nutritional status factors and frailty in community-dwelling older Korean adults. Our findings highlight the aspects to be considered during screening, educating, and in counseling programs that aim to reduce incidence of nutrition-related frailty in community-dwelling older adults.

Regarding our first aim, according to the results, 32.9% and 1.7% of the older adults who were robust became pre-frail and frail, respectively, after two years. Similar to our study, according to a systematic review of cohort studies among community-dwelling older adults ≥ 60 years using any frailty diagnostic criteria for identification [11], 30.9% of previously robust older adults became pre-frail after a median 2.5 years of follow-up. In particular, considering that only 3% of frail older adults ≥ 60 years living in the community became robust based on the frailty phenotype criteria [25], the incidence of frailty is very high among robust older adults living in the community. Therefore, it is important to design interventions to increase awareness of factors that increase the risk of frailty and of ways to minimize that risk.

According to the results, regarding our second aim, this study found a significant longitudinal association between specific nutritional status factors such as anorexia, psychological stress, acute disease, and BMI at T1, and the incidence of pre-frailty or frailty at T2, even after adjusting for general characteristics. However, this study did not find a significant longitudinal association between malnutrition, or the risk of malnutrition, and frailty compared to normal nutrition, which is contrary to previous cross-sectional studies where nutritional status was associated with frailty [12]. This could be because of time considerations. Wei et al. [26] showed that robust malnourished older adults developed frailty after a five-year follow-up (AOR, 3.45; 95% CI, 1.00–11.9). According to the cycle of malnutrition and frailty phenotype proposed by Fried et al. [5], malnutrition causes negative energy balance and weight loss, resulting in sarcopenia, decreased physical activity, and increased disability and dependence. Therefore, malnutrition takes a long time to affect frailty. However, because our study was only conducted over two years, nutritional status could not affect frailty. This result indicates the need to identify longitudinal associations over a longer period for all community-dwelling older adults.

The observed association between anorexia and frailty is consistent with the findings of previous studies [27–29]. Evidence indicates that anorexia is a predictor of malnutrition, frailty, disability, and mortality in older adults [27–29]. Proper nutritional evaluation is essential for the integrated evaluation of older adults; in particular, it is necessary to identify the causes or consequences of anorexia [28]. Anorexia in older adults was described in two categories in a scoping review: physiological dysfunctions related to pathologies and polypharmacy, and non-physiopathological dysfunctions related to psychological, sociocultural, and environmental problems [30]. As non-physiopathological problems are potentially modifiable, they may be an appropriate avenue for interventions to prevent and manage anorexia [30]. The prevention and management of anorexia, involves food manipulation, modification of environmental risk factors, and treatment of pharmacological and medical problems [31]. A randomized controlled trial that provided older adults with solid nutritional supplements in consideration of appetite, edentulous status, and appropriate texture confirmed positive effects on weight gain and appetite increase [32]. Therefore, early detection of older adults at risk for anorexia, and the provision of subsequent interventions that have been confirmed to be effective in previous studies, can help prevent frailty.

The MNA-SF assesses psychological stress and acute diseases and can identify psychological frailty, including cognitive functioning, mood, and motivational components, which are important in determining the state of frailty [33, 34]. Frail older adults tend to suffer from anxiety, depression, and acute diseases [35–37]. An increase in psychological distress, such as anxiety and depression, in older adults has negative consequences for nutrition, frailty, disability, and death [36–38]. Psychological distress factors are associated with hyposalivation, which has been confirmed to be associated with anorexia [39, 40]. Acute diseases experienced by older adults have also been shown to affect nutrition and frailty [41, 42]. These findings require further exploration related to the effect of psychological distress and acute diseases on the nutritional status and frailty in older adults.

Weight loss is a major component of other phenotypic definitions of frailty [43]. In this study, older adults with a low BMI (< 19) had a higher risk of pre-frailty or frailty than those with a BMI (≥ 23) (AOR, 4.11; 95% CI, 1.20–14.04). A recent systematic review and meta-analysis showed that being underweight (BMI < 18.5) was associated with an increased risk of frailty (relative risk [RR], 1.45; 95% CI, 1.10–1.90) [44]. However, obesity is also a risk factor for frailty; BMI (≥ 30) was associated with a higher risk (RR, 1.40; 95% CI, 1.17–1.67) and confirmed the U-shaped risk relationship between BMI and frailty [44]. This study confirmed that the risk of pre-frailty or frailty was higher among those who were underweight than among those who were obese. However, considering that BMI and frailty have U-shaped patterns, future studies are needed to subdivide the degree of BMI and to confirm the risk relationship between frailty and BMI.

For the prevention and management of frailty among community-dwelling older adults, it is necessary to comprehensively consider not only individual general and health risk factors [45] but also nutrition-specific risk factors. Japan, which has rapidly changed into a super-aged society, has already prepared detailed food standards and guidelines [46]. It successfully conducted a lunch box project for community-dwelling older adults by identifying nutritional risk factors, such as frailty status, number of teeth, chewing, swallowing function, and the presence or absence of a caregiver [46]. Similarly, since July 2020, Korea has been conducting a pilot project for customized meal support and nutrition management services for community-dwelling low-income older adults as part of the community-integrated health promotion project [47]. This project considers physical function, oral function such as chewing and swallowing, and nutritional status through health screening for older adults [47]. These projects [46, 47], which comprehensively evaluate individual risk factors, are customized and practical nutritional evaluations and frailty intervention strategies. Considering the close association between malnutrition, frailty, and adverse health outcomes, early identification and prevention of nutritionally associated risk factors plays a crucial role in health policymaking. However, as anorexia diagnosis in older adults focuses on weight loss, BMI, and oral intake rather than appetite assessment [48], a standardized assessment that includes appetite, food taste, digestion, and oral function is needed in the future.

This study has several limitations. First, the sample of this study included individuals living independently in the community who could ambulate to visit the center, and excluded home-bound disabled or institutionalized older adults and dementia patients with communication problems. Therefore, it is possible that participants with a relatively low probability of frailty were recruited. In addition, according to the MNA-SF, malnutrition, the risk of malnutrition, and normal nutritional status were 0.2%, 9.4%, and 90.4% for all participants, respectively. The distribution of nutritional status indicated a significant difference between groups, which may have weakened the effect size between nutritional status and frailty. Future studies should focus on extensive primary data integration that consider residents of nursing homes or facilities in the community to compare and predict the results. Second, our results were derived from a secondary data analysis. The KFACS only recruited older adults aged 70–84 years due to the relatively small frailty prevalence rate of Korean adults aged 65–69. In addition, it was also relatively difficult for older adults aged 85 or older to visit the center and complete follow-up surveys [7, 49]. Since the prevalence of frailty can be different worldwide, comparative studies that included adults aged 65–69 years are required. In contrast, considering that frailty deteriorates as age increases, a future study is required to predict the longitudinal association of nutritional status and frailty through re-analysis that includes older adults aged 85 years and older. Nevertheless, this study was a large, population-based study of older adults to investigate the longitudinal association between nutritional status and frailty in Korea. Third, nutritional status was assessed using the self-report questionnaire of the MNA-SF; therefore, there may be problems with reporting and/or recall bias. Specifically, it should be noted that weight loss and immobility of the MNA-SF are closely associated domains of the frailty phenotype [50]. In the future, validation through an additional objective investigation method will be required. Nevertheless, the MNA-SF is the most validated nutritional screening tool for older adults, and this study included blood nutritional biomarkers as objective data. Finally, this study did not consider the cross-sectional effects of nutritional status and covariates at T2 on the incidence of frailty at T2. Hence, further studies are required with repeated measures analysis that include gradual changes in nutritional status and covariates to confirm the potential causal relationship as well as longitudinal association between nutritional status and frailty. These studies are expected to be possible when data above the third wave is released.

## Conclusions

This study investigated the incidence of pre-frailty or frailty in 34.6% of the participants in a longitudinal study of older adults living in a community over a two-year period. Specifically, this study found that anorexia, psychological stress, acute disease, and BMI were significant longitudinal factors associated with frailty. The specific risk factors of MNA-SF identified in this study can be used to develop screening, customized nutrition education, and counseling programs to reduce the incidence of nutrition-related frailty in community-dwelling older adults.

## Supplementary Information

**Additional file 1.**

## Data Availability

The data that support the findings of this study are available from the KFACS but restrictions apply to the availability of these data, which were used under license for the current study, and so are not publicly available. Data are however available from the corresponding authors (NK) upon reasonable request and with permission of KFACS.

## Abbreviations

ADL: Activities of daily living
AOR: Adjusted odds ratio
BMI: Body mass index
CI: Confidence interval
KFACS: Korean Frailty and Aging Cohort Study
IADL: Instrumental activities of daily living
IRB: Institutional Review Board
MNA-SF: Mini Nutritional Assessment Short-Form
OR: Odds ratio
RR: Relative risk
