# Supplementary material for: Two-year longitudinal associations between nutritional status and frailty in community-dwelling older adults: Korean Frailty and Aging Cohort Study
Source: BMC Geriatr. 2023 Apr 5;23:216. doi: 10.1186/s12877-023-03903-4 (PMC10074647; doi:10.1186/s12877-023-03903-4)
Supplement: Supplementary file 1 — Additional file 1. [file 12877_2023_3903_MOESM1_ESM.docx]

**(a) Mini Nutritional Assessment**

**(b) Anorexia**

**(c) Weight Loss**

**(d) Psychological Stress or Acute Disease**

**(e) Neuropsychological Problems**

**(f) BMI**

**Supplementary Material Fig. S1** Incidence of pre-frailty or frailty two-year later according to baseline nutritional status.

Note: Frailty is categorized into robust and non-robust (pre-frail or frail) group.

BMI, body mass index.
